# Sugar Is the Key Cause of Overweight/Obesity in Sugar-Sweetened Beverages (SSB)

**DOI:** 10.3389/fnut.2022.885704

**Published:** 2022-06-28

**Authors:** Lianlong Yu, Han Zhou, Fengjia Zheng, Jian Song, Yutong Lu, Xiao Yu, Changsheng Zhao

**Affiliations:** ^1^Shandong Center for Disease Control and Prevention, Jinan, China; ^2^Queen Mary School, Nanchang University, Nanchang, China; ^3^Department of Nutriology, The Second Hospital of Shandong University, Jinan, China

**Keywords:** children, adolescents, overweight, obesity, sugar-sweetened beverages

## Abstract

To evaluate association between overweight/obesity and sugar-sweetened beverages (SSB) types, SSB sugar, among children and adolescents. A total of 1,068 children and 751 adolescents were identified from a provincial survey conducted in Shandong, China. *χ^2^* tests, logistic regression, restricted cubic spline (RCS), mediation analysis, pathway analysis, and ordinary least square (OLS) regression were applied to test association among overweight/obesity, SSB sugar, SSB types, and a set of factors. The mean daily SSB intake for children and adolescents was 210.7 and 208 ml, respectively. The threshold of SSB sugar consumption causing overweight/obesity was around 25 g/day and verified by RCS based on logistic regression. Approximately, 44.8% of the study subjects consumed more than 25 g/day of SSB sugar. SSB sugar intake above 25 g/day resulted in higher risk of overweight/obesity (OR = 1.391, 95% CI, 1.115–1.734). The effects of all types of SSBs on overweight/obesity were fully mediated by SSB sugar (*p* < 0.05), except for milk. Consumption of any types of SSBs had a positive impact on SSB sugar intake in both children and adolescents. Any type of SSB intake was a risk factor in excessive intake of SSB sugar (OR > 1, *p* < 0.05). In particular, milk powder, tea, and tea-flavored drinks, and carbonated have greater ORs for excessive intake of SSB sugar (OR = 76.08, 8.879, 4.355, *p* < 0.05, respectively). It was found that the effect of SSB on overweight/obesity was mediated by the intake of SSB sugar, and the effects of various SSBs were different according to multiple linear regression and pathway analysis (*p* < 0.05, respectively).

## Introduction

The prevalence of obesity in children and adolescents was increasing year by year in countries around the world, and the burden of various chronic diseases caused by this has become a serious public health problem (1). More than 380 million children and adolescents worldwide were suffering from obesity and related chronic diseases (2). Shandong Province was a coastal province in eastern China. As of 2020, the total population of Shandong has reached 101.06 million. Worryingly, the prevalence of overweight and obesity among children and adolescents in Shandong Province was 15.05 and 9.23%, respectively, in (3). According to previous reports in the literature, consumption of sugar-sweetened beverages (SSB) may be one of the causes of overweight and obesity (4–6). Some papers have also shown that high SSB intake was strongly associated with youth overweight and obesity (7–10). In the past years, SSB intake has increased among United States youth (11), which has become a major source of calories in the diets (12). The daily consumption of SSBs among people over 1 year old in South Korea increased from an average of 86.6 grams to 184.5 grams from 2008 to 2018, and the consumption more than doubled in 10 years (13). In the past decades, the SSBs consumption in Chinese children and adolescents has increased significantly. Meanwhile, SSBs were risk factors in obesity and hypertension in children and adolescents. In recent years, the prevalence of obesity and hypertension among Chinese children and adolescents has also increased (14).

On the contrary, some of the studies focused on contributing calories of the SSB (15). Because they believed that the main reason SSB cause overweight/obesity was providing body with surplus energy. Nevertheless, the dose-response relationship between overweight/obesity and SSB sugar intake has been less investigated in children and adolescents. The threshold at which SSBs sugar contributes to overweight and obesity also needs to explore. It was also unclear whether SSBs sugar played a dominant role in causing overweight/obesity. Meanwhile, the contribution of different types of SSB to the risk of excess sugar was also an issue that needs to be clarified. All of these would guide SSB types choice of children and adolescents.

Therefore, the objectives of this study were to investigate the relationship between overweight/obesity SSBs and SSB sugar, and to explore whether SSBs affect overweight/obesity directly or mediated through SSB sugar, and whether the type of SSB affects SSB sugar intake and overweight/obesity in children (6–12 years) and adolescents (13–18 years old).

## Materials and Methods

### Study Design and Population

The data for this study were derived from the SSB consumption data collected by the Shandong Provincial Center for Disease Control and Prevention from 2013 to 2016. The data were based on a cross-sectional survey of multistage stratified regional probability sampling of individual children aged 6–18. Samples were drawn from ten cities in Shandong Province using a hierarchical multistage clustering random process. The investigation cities included Jinan, Qingdao, Dongying, Jining, Laiwu, Liaocheng, Linyi, Taian, Weifang, and Yantai. According to geographical location, eating habits, and economic conditions, 10 survey sites were selected in Shandong Province, primary and secondary schools were randomly sampled, and classes were randomly sampled according to grade distribution. The male-to-female ratio was 1:1, and the urban-rural population ratio was 1:2. The final study population included 1,068 children aged 6–18 and 751 adolescents, which included SSB intake and height and weight data. This study was reviewed by the Ethics Committee of the Shandong Provincial Center for Disease Control and Prevention.

### Definitions

Sugar-sweetened beverages were defined as beverages that contain added caloric sweeteners, such as sugar or high-fructose corn syrup (16, 17). According to the food category in FCT (food composition table) 2002/2004 and actual situation in the Chinese market, SSBs were classified as follows: carbonated, fruit vegetable juice, vegetable protein drink, tea and tea-flavored drinks, coffee, milk (containing added sugar), milk powder, yogurt, milk-containing drinks and cheese, all of which containing added sugar.

### Measurements

The 24-h SSB recall method was used to estimate SSB consumption levels among 6–18 years old children. A 24-h recall for 3 days was performed on each subject by uniformly trained investigators. The investigation was completed in the presence of the children’s guardians. At the same time, for children with insufficient cognitive ability, their guardians completed the information response. In this survey, weight was measured using a uniform and calibrated scale, the subjects were asked to wear light clothing, measurements were accurate to 0.1 kg, height was measured using a portable range finder, the subjects were asked to wear no shoes, and measurements were accurate to 0.1 cm. BMI was equal to weight (kg) divided by height (meters) squared. In this study, height and weight were measured using uniformly purchased medical-grade equipment, and the investigators were uniformly trained medical staff. For *χ^2^* tests, three weight status categories (normal, overweight, and obesity) were classified based on the Chinese standard reported by Group of China Obesity Task Force (18). For logistic regression analysis, a positive outcome for the outcome variable was defined as being overweight or obesity. SSB intake was dichotomized by ≥ 200 ml/day based on SSB consumption distribution and daily SSB consumption packaging units. And SSB sugar intake was dichotomized by ≥ 25 g/day based on the suggestion of “Dietary Guidelines for Chinese Residents” (19).

### Variables

Demographically, SSB composition and types of SSB were gathered as exposure variables. Demographic factors included age (6–18 years), gender, region (urban and rural), and weight status. The weight status of children and adolescents was divided using the unified Chinese standard “China’s Growth Chart,” which was based on the age- and gender-specific body mass index (BMI, kg/m^2^) distribution of the large population, and the weight status was divided into normal weight (≥5 to <85%), overweight (≥85 to <90%), and obese (≥90%) (18). Consumption variables included SSB intake of various categories. Analysis of SSB sugar intake was based on Chinese FCT 2002/2004 and the China FCD system.

### Statistical Analysis

Chi-square tests were used to compare the percentage of gender, child and adolescents, and region in different weight statuses (normal, overweight, and obesity). Odds ratios (ORs) and 95% confidence intervals (CIs) for overweight/obesity-related variables were estimated by logistic regression models. Multivariable logistic regression models were adjusted for possible confounding factors: gender, age, region, SSB intake, SSB categories. The relationships between SSB sugar intake and SSB categories were assessed by multiple linear regression models, adjusting for sex, age, and region. Restricted cubic spline (RCS) based on logistic regression equation models was used to evaluate the associations between levels of SSB sugar intake and overweight/obesity. Mediation analysis and pathway analysis were utilized to assess the relationship among SSB consumption, SSB sugar intake, and overweight/obese. R4.1.2 was used for data statistical analyses and graphing. *P*-value < 0.05 was considered statistically significant.

## Results

**FIGURE 1:**
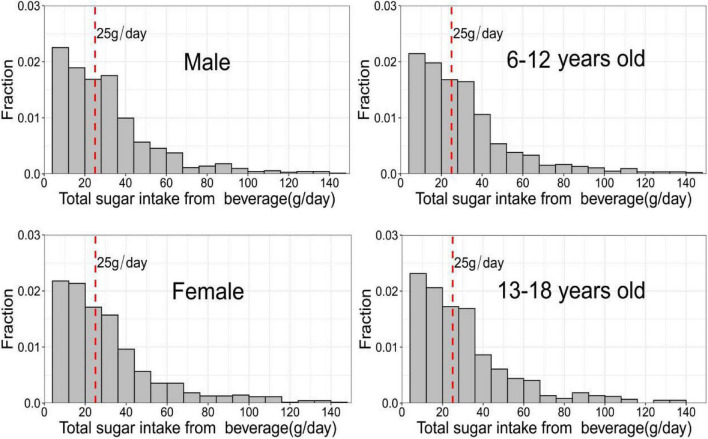
Sugar intake from beverages in different age and gender groups.

The average of SSB consumption among 6–18-year-old children and adolescents was 209.6 ml/day, 203.8 ml/day for males, 215.4 ml/day for females, 210.7 ml/day for those aged 6–12 years, and 208 ml/day for those aged 13–18 years. Overall, SSB intake for each quartile was 60.5 ml/day for Q1, 152.1 ml/day for Q2, 278.6 ml/day for Q3, and 450 ml/day for the 90th percentile. Sugar intake (from SSB) of ≥ 25 g/day was found in 44.8% of the children and adolescents. Figure 1 shows the intake of SSB sugar in different gender and age groups. Table 1 shows demographic characteristics and weight status (normal, overweight, and obesity). Based on *χ^2^* tests, there were differences in the distribution of weight status among different types of gender, age groups, and regions. Table 2 showed the consumption of different types of beverages by children and adolescents.

**TABLE 1:** Characteristics of study subjects and their associations with weight status among 6–18 years in Shandong, China.

Characteristic	All		Weight classification*						χ ^2^†	*P*
			Normal		Overweight		Obese			
	*N*	(Weighted, %)	*N*	(Weighted, %)	*N*	(Weighted, %)	*N*	(Weighted, %)		
No. of participants	1819	100.0	1379	75.8	123	6.8	317	17.4		
**Gender**										
Boys	916	50.4	653	71.3	62	6.8	201	21.9	25.4714	<0.0001
Girls	903	49.6	726	80.4	61	6.8	116	12.8		
**Age group**										
6–12 year	1068	58.7	764	71.5	76	7.1	228	21.3	29.3638	<0.0001
13–18 year	751	41.3	615	81.9	47	6.3	89	11.9		
**Region**										
Urban	522	28.7	354	67.8	43	8.2	125	23.9	26.0575	<0.0001
Rural	1297	71.3	1025	79.0	80	6.2	192	14.8		
BMI, kg/m^2^[mean (SE)]	19.0	3.9	17.5	2.5	20.9	2.9	24.3	4.2		

†χ^2^ tests were used for each variable to examine differences across categories; *measured weight and height were used to calculate body mass index (BMI). Underweight was defined as BMI < 5th percentile; normal weight was defined as BMI ≥ 5th to < 85th percentile; overweight was defined as BMI ≥ 85th to < 90th percentile; and obesity was defined as BMI ≥ 90th percentile based on age- and gender- specific reference data from the Chinese growth charts.

**TABLE 2:** Consumption of various types of SSBs in children and adolescents (mean ± SD).

	6–12 year (*n* = 1068)	13–18 year (*n* = 751)	*t*	*P*
Carbonated	19.2 ± 42.2	20.6 ± 43.4	−0.66	0.512
Fruit vegetable juice beverage	14.5 ± 50.1	12.4 ± 31.3	1.09	0.2739
Vegetable protein drinks	16.6 ± 39.1	20.0 ± 39.7	−1.8	0.0727
Tea and tea flavored drink	6.9 ± 25.8	9.2 ± 35.3	−1.52	0.1277
Coffee	2.7 ± 19.6	4.2 ± 25.7	−1.37	0.1712
Milk	82.3 ± 120.8	72.1 ± 123	1.77	0.0777
Milk powder consumption, g/day	1.6 ± 12.3	1.5 ± 9.6	0.17	0.8659
Yogurt	54.7 ± 90.3	52.6 ± 88.6	0.50	0.6180
Milk containing drink	10.2 ± 31.6	13.6 ± 36.7	−2.05	0.0406
Cheese	1.8 ± 17.3	1.7 ± 11.0	0.15	0.8787

*Two-sample t-test was used for comparison between two groups.*

**FIGURE 2:**
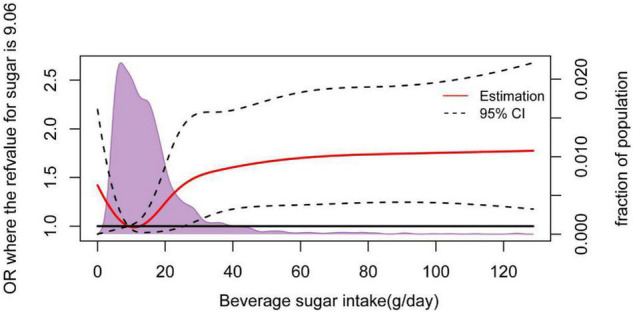
An adjusted odds ratio for beverage sugar intake and overweight/obesity using restricted cubic spline (RCS) analysis. The RCS model adjusted for age, gender, and region. The solid red line is the multivariate adjusted hazard ratio, and the dashed line shows the 95% confidence interval from restricted cubic spline regression. The unassociated reference line is shown in solid bold based on the basic and beverage consumption data, gathered by the Shandong Center for Disease Control and Prevention from 2013 to 2016.

Meanwhile, based on the *t*-test, SSB sugar intake, total SSB consumption, and some kinds of SSB consumption differed significantly by weight classification (data not shown). As shown in Table 3, there were differences in the results of univariate and multivariate logistic regression analyses. In univariate logistic regression results, the OR for overweight/obesity was significantly different by gender, age group, region, summary SSB intake, fruit and vegetable juice intake, vegetable protein beverage intake, and sugar intake (from SSB). However, the adjusted OR for overweight/obese in multivariable logistic regression analysis did not differ significantly by intake of fruit and vegetable juice and vegetable protein drinks. The RCS using P25 (9.06 g/day) of SSB sugar intake as the reference line showed that the risk of overweight/obesity was increased when the intake was > 25 g (Figure 2).

**TABLE 3:** Adjusted odds ratios for variables associated with overweight/obese among 6–18 years in Shandong, China.

Characteristic	Overweight/obese			
	Model 1†: univariate		Model 2‡:multivariate	
	Adjusted OR	95%CI	Adjusted OR	95%CI
**Gender**				
Boys	1.00 (reference)		1.00 (reference)	
Girls	0.605*	0.487–0.753	0.586*	0.469–0.731
**Age group**				
6–12 year	1.00 (reference)		1.00 (reference)	
13–18 year	0.556*	0.442–0.698	0.542*	0.430–0.684
**Region**				
Urban	1.00 (reference)		1.00 (reference)	
Rural	0.559*	0.445–0.702	0.573*	0.454–0.722
**SSB intake**				
<200 ml/day	1.00 (reference)		1.00 (reference)	
≥200 ml/day	1.449*	1.168–1.798	1.384*	1.110–1.726
**Beverage classification, ml[mean (SE)]**				
**Carbonated**				
<200 ml/day	1.00 (reference)		1.00 (reference)	
≥200 ml/day	1.188	0.863–1.637	0.758	0.959–6.963
**Fruit vegetable juice beverage**				
<200 ml/day	1.00 (reference)		1.00 (reference)	
≥200 ml/day	2.651*	1.137–6.177	2.584	0.959–6.963
**Vegetable protein drinks**				
<200 ml/day	1.00 (reference)		1.00 (reference)	
≥200 ml/day	2.184*	1.006–4.743	1.911	0.769–4.753
**Tea and tea flavored drink**				
<200 ml/day	1.00 (reference)		1.00 (reference)	
≥200 ml/day	1.751	0.584–5.252	0.975	0.269–3.532
**Coffee**				
<200 ml/day	1.00 (reference)		1.00 (reference)	
≥200 ml/day	3.159	0.910–10.962	4.098	0.981–17.131
**milk**				
<200 ml/day	1.00 (reference)		1.00 (reference)	
≥200 ml/day	1.118	0.852–1.468	1.026	0.774–1.358
**milk powder**				
<10 g/day	1.00 (reference)		1.00 (reference)	
≥10 g/day	0.743	0.382–1.448	0.558	0.274–1.138
**yogurt**				
<200 ml/day	1.00 (reference)		1.00 (reference)	
≥200 ml/day	0.998	0.695–1.432	0.917	0.630–1.334
**milk containing drink**				
<200 ml/day	1.00 (reference)		1.00 (reference)	
≥200 ml/day	1.257	0.485–3.260	0.544	0.165–1.786
**cheese**				
<10 g/day	1.00 (reference)		1.00 (reference)	
≥10 g/day	1.555	0.901–2.683	1.400	0.792–2.474
**sugar intake (from beverage)**				
<25 g/day	1.00 (reference)		1.00 (reference)	
≥25 g/day	1.455*	1.173–1.805	1.391*	1.115–1.734

*A significant finding based on the 95% confidence interval (CI); †Model 1 did not include all variables of study. The reference category included subjects with overweight and obesity. Measured weight and height were used to calculate body mass index (BMI). Underweight was defined as BMI < 5th percentile; normal weight was defined as BMI ≥ 5th to < 85th percentile; overweight was defined as BMI ≥ 85th to < 90th percentile; and obesity was defined as BMI ≥ 90th percentile based on age- and gender- specific reference data from the Chinese growth charts. ‡Model 2 included all variables of study. The reference category included subjects with overweight and obesity.

**FIGURE 3:**
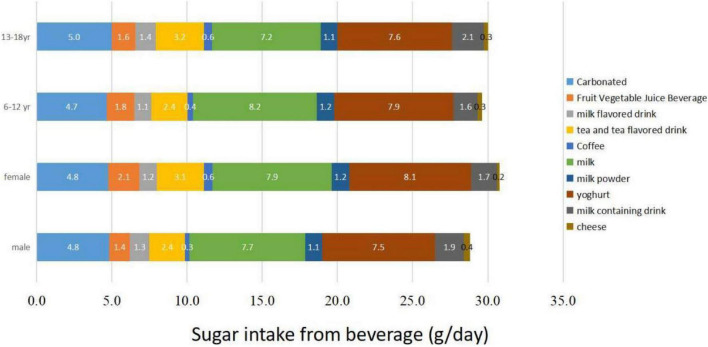
Beverage sources of sugar in different age and gender groups.

According to the food composition database source from Chinese FCT 2002/2004 and the China FCD system, the mean sugar content of carbonated, fruit vegetable juice, vegetable protein drinks, tea and tea-flavored drinks, coffee, milk (containing added sugar), milk powder, yogurt, milk-containing drinks, and cheese was 13.8 g/100 ml, 9.1 g/100 ml, 5.9 g/100 ml, 10.7 g/100 ml, 10.5 g/100 ml, 5.9 g/100 ml, 58.4 g/100 g, 11.4 g/100 ml, 11.4 g/100 ml, 12.6 g/100 ml. Among them, the sugar intake from milk, yogurt, and carbonated drinks was higher, and there were different types of SSBs consumption in different age groups and types of gender. Figure 3 shows sugar sources in SSBs for different age and gender groups.

Table 4 shows the consumption of different SSB categories for children and adolescents and the relationship between different SSB categories and total SSB sugar intake. The relationships were tested using multiple linear regression models in this table. Based on the linear regression model adjusted for sex, BMI and region, the consumption of carbonated, fruit vegetable juice, vegetable protein drinks, tea and tea-flavored drinks, coffee, milk, milk powder, yogurt, milk-containing drinks, and cheese has positive effect on sugar intake (a source from SSB) both in children and adolescents. As shown in Table 5, all types of SSB intake were risk factors in excessive SSBs sugar intake (≥25 g/day) in children and adolescents (OR > 1, *p* < 0.05).

**TABLE 4:** Relationships between sugar intake from SSB and different types of beverage consumption among 6–18 years in Shandong, China.

Types of SSB	*N*	Mean	Standard deviation	P50	Q1	Q3	Q Range	Partial regression coefficient	*P*
**Carbonated consumption, ml/day**									
6–12 year	1068	19.2	42.2	2.1	0	21.4	21.4	0.24	<0.0001
13–18 year	751	20.6	43.4	6.6	0	28.6	28.6	0.24	<0.0001
**Fruit Vegetable Juice Beverage consumption, ml/day**									
6–12 year	1068	14.5	50.1	0	0	10.5	10.5	0.13	<0.0001
13–18 year	751	12.4	31.3	0	0	10	10	0.13	<0.0001
**vegetable protein drinks consumption, ml/day**									
6–12 year	1068	16.6	39.1	0	0	15	15	0.07	<0.0001
13–18 year	751	20	39.7	0	0	28.6	28.6	0.07	<0.0001
**tea and tea flavored drink consumption, ml/day**									
6–12 year	1068	6.9	25.8	0	0	0.4	0.4	0.35	<0.0001
13–18 year	751	9.2	35.3	0	0	1.1	1.1	0.35	<0.0001
**Coffee consumption, ml/day**									
6–12 year	1068	2.7	19.6	0	0	0	0	0.13	<0.0001
13–18 year	751	4.2	25.7	0	0	0	0	0.13	<0.0001
**milk consumption, ml/day**									
6–12 year	1068	82.3	120.8	28.6	0	137.1	137.1	0.1	<0.0001
13–18 year	751	72.1	123	16.7	0	100	100	0.1	<0.0001
**milk powder consumption, g/day**									
6–12 year	1068	1.6	12.3	0	0	0	0	0.73	<0.0001
13–18 year	751	1.5	9.6	0	0	0	0	0.73	<0.0001
**yogurt consumption, ml/day**									
6–12 year	1068	54.7	90.3	21.4	0	67.1	67.1	0.15	<0.0001
13–18 year	751	52.6	88.6	17.9	0	71.4	71.4	0.15	<0.0001
**milk containing drink consumption, ml/day**									
6–12 year	1068	10.2	31.6	0	0	6	6	0.16	<0.0001
13–18 year	751	13.6	36.7	0	0	6.9	6.9	0.16	<0.0001
**cheese consumption, g/day**									
6–12 year	1068	1.8	17.3	0	0	0	0	0.16	<0.0001
13–18 year	751	1.7	11	0	0	0	0	0.16	<0.0001

*The relationships among sugar intake and beverage categories were assessed by multiple linear regression models adjusted for sex, region, and consumption of various beverages.*

**TABLE 5:** Logistic regression analysis of relationship between excessive SSB sugar intake (≥25 g/day) and consumption of intake of various beverages.

	OR	95% CI		*P*
SSB intake	1.044	1.039	1.049	<0.0001
age	1.063	0.999	1.132	0.0523
sex	0.852	0.562	1.293	0.4527
region	0.687	0.432	1.091	0.1113
Carbonated (ml/day)	4.355	2.191	8.655	<0.0001
Fruit vegetable juice beverage (ml/day)	2.237	1.511	3.314	<0.0001
vegetable protein drink (ml/day)	1.544	1.258	1.896	<0.0001
tea and tea flavored drink (ml/day)	8.897	2.857	27.708	0.0002
Coffee (ml/day)	2.553	1.605	4.062	<0.0001
milk (ml/day)	1.859	1.393	2.482	<0.0001
milk powder (g/day)	76.08	9.54	606.704	<0.0001
yogurt (ml/day)	2.405	1.601	3.614	<0.0001
milk containing drink (ml/day)	2.513	1.604	3.936	<0.0001
cheese (ml/day)	2.736	1.281	5.847	0.0094

*Logistic regression is adjusted for variables age, sex, region, and consumption of various beverages.*

**FIGURE 4:**
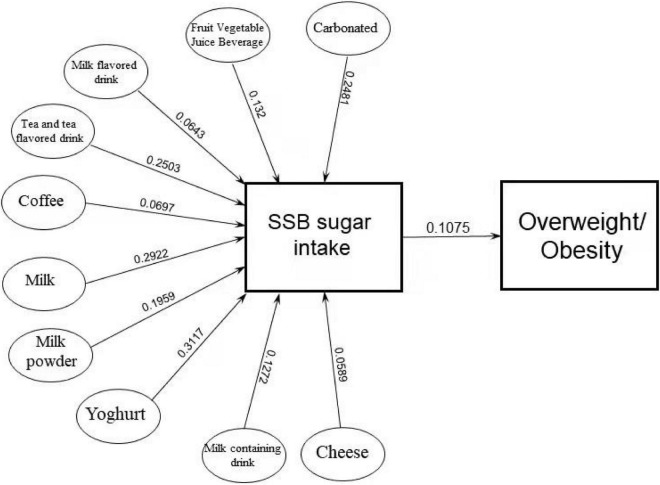
Standardized estimation of the relationship of types of SSB, SSB sugar intake, and overweight/obesity.

The direct effects of all types of SSBs on overweight/obesity were not statistically significant (*p* > 0.05), except for milk. The indirect effects of all types of SSBs on overweight and obesity through sugar were statistically significant (*p* < 0.05). By testing for mediating effects, we found that sugar exerted a full mediating effect on overweight/obesity (*p* < 0.05) in all types of SSB, except for milk. Figure 4 shows the path analysis diagram with standardized estimates for the relationship between the types of SSB, SSB sugar intake, and overweight/obesity. The one-sided arrows from different types of SSB sugar intake to overweight/obesity represent the regression coefficients; SSB sugar intake was positively correlated with overweight/obesity in children and adolescents (β = 0.1075, *p* < 0.05).

## Discussion

In a previous study (20, 21), the prevalence of overweight/obesity was increasing significantly both in urban and rural areas from 1985 to 2014 in Shandong, China. The prevalence of overweight and obesity increased from less than 1% in 1985 to nearly 20% in 2014. In our study, the overweight and obesity status among children and adolescents, the rural area was more serious than the urban area significantly (*p* < 0.0001). Meanwhile, the overweight and obesity status in boys and children were more serious, respectively, than girls and adolescents (*p* < 0.0001). Girls were associated with a lower OR for overweight and obesity (OR = 0.586). Similar phenomena have been observed in adolescents (OR = 0.542) and the rural area (OR = 0.573). These findings were similar to studies of the weight status of children and adolescents across China (22, 23).

The consumption of SSBs has been reported as a risk factor in overweight and obesity in previous reviews (24–26). Reducing SSB consumption among obese and overweight youth may help to curb childhood obesity (27). On the contrary, some of the studies were not designed to evaluate whether SSB contributes to overweight and obesity (27–29) other than their role in contributing calories. In their views, it was unclear whether SSB intake, as part of current diet, was associated with obesity, and, if so, whether this association was because SSB was a significant contributor to calories, or because of other reasons that may be related to SSB consumption [e.g., socioeconomic status (30, 31), physical condition (32)].

In our study, high SSB intake (≥200 ml/day) was associated with a higher OR for overweight and obesity (OR = 1.384, *p* < 0.05), and SSB sugar intake (OR = 1.391, *p* < 0.05) (Table 3). This was in agreement with studies that were reported by Brunkwall L, Hu Fb, and Bremer AA (24, 33, 34). RCS results suggest that SSB sugar intake of more than 25 g/day increased the risk of overweight/obesity, which was consistent with the dietary guidelines for Chinese residents, recommending that sugar intake should not exceed 25 g/day.

In our discovery, the effects of all types of SSBs on overweight/obesity were fully mediated by SSB sugar, except for milk. This explains why previous studies have shown that different types of beverages have different effects on overweight/obesity. In previous studies, the consumption of tea, milk-flavored beverages, and fruit and vegetable juice beverages was not associated with overweight and obesity (35–38). Similarly, Junichi R Sakaki’s study also found that the consumption of orange juice was well correlated with height growth, but not with excessive weight gain (39). Additionally, few studies have tested the relationships between overweight/obesity and SSB sugar intake. Meanwhile, few studies mentioned the contribution of SSB categories to the sugar intake. In order to test the sugar intake from SSB, we gathered the data of sugar content in SSB from Chinese FCT 2002/2004 and the China FCD system. In our study, consumption of any type of SSB had a positive effect on SSB sugar intake in children and adolescents, suggesting that consumption of any type of SSB could cause an increasing SSB sugar intake.

The results of mediation analysis and pathway analysis showed that sugar exerted a full mediating effect on overweight/obesity (*p* < 0.05) in all types of SSB, except for milk. The intake of different types of SSBs could cause the increase of SSB sugar intake, and the increase of SSB sugar intake was a risk factor in overweight/obesity in children and adolescents. The results of logistics regression analysis showed that any type of SSB intake was a risk factor in excessive intake of SSB sugar (≥25 g/day) (OR > 1, *p* < 0.05). This implied that excessive consumption of sugary beverages, especially sugar, was a risk factor in overweight/obesity in children and adolescents. In previous studies (40), some scholars have also encouraged children to use plain water instead of SSB consumption, because higher SSB sugar intake might lead to excess calories in the diet, and SSB sugar intake itself was an adverse factor in reducing overall dietary quality. The strength of our study was to clarify that the reason for the overweight/obesity of SSBs was entirely due to the sugar in SSBs, not other components. At the same time, we also analyzed the risk of excessive SSB sugar intake caused by various beverages (Table 5).

## Conclusion

According to the consumption of SSB drinks in Shandong province from 2013 to 2016, the average daily SSB intake was 210.7 and 208 ml in children and adolescents, respectively. The effects of all types of SSBs on overweight/obesity were fully mediated by SSB sugar, except for milk. There might be a dose-response relationship between overweight/obesity and SSB sugar intake, and daily intake of SSB sugar over 25 g was a risk factor in overweight/obesity in children and adolescents. There were 44.8 percent of children and adolescents, consuming 25 grams or more of sugar per day. SSB sugar intake, which was a harmful factor in overweight/obesity, was influenced by the SSB types significantly. Since the consumption of any type of SSB had a positive effect on SSB sugar intake and any type of SSB intake was a risk factor in excessive SSB sugar intake, we recommend reducing the consumption of all types of SSBs, especially those with a higher sugar content.

The main limitation of our study was the lack of data in dietary energy intake and physical activity among these children and adolescents. We tried to verify their impact with pre-experiments and found that the impact was limited and did not affect the trend of the results. We hope that future studies to be conducted will address this area.

## Data Availability Statement

The datasets presented in this article are not readily available because the copyright of the dataset is currently owned by the Shandong Center for Disease Control and Prevention and The Second Hospital of Shandong University, and has not been fully disclosed yet.

## Author Contributions

LY and CZ: conceptualization. LY and HZ: formal analysis. LY, FZ, and JS: investigation. LY, HZ, and FZ: methodology. LY, YL, and XY: validation. LY: writing – original draft preparation. HZ and CZ: writing – review, and editing. All authors contributed to the article and approved the submitted version.

## Conflict of Interest

The authors declare that the research was conducted in the absence of any commercial or financial relationships that could be construed as a potential conflict of interest.

## Publisher’s Note

All claims expressed in this article are solely those of the authors and do not necessarily represent those of their affiliated organizations, or those of the publisher, the editors and the reviewers. Any product that may be evaluated in this article, or claim that may be made by its manufacturer, is not guaranteed or endorsed by the publisher.
